# Variation of DNA Methylome of Zebrafish Cells under Cold Pressure

**DOI:** 10.1371/journal.pone.0160358

**Published:** 2016-08-05

**Authors:** Bingshe Han, Wenhao Li, Zuozhou Chen, Qiongqiong Xu, Juntao Luo, Yingdi Shi, Xiaoxia Li, Xiaonan Yan, Junfang Zhang

**Affiliations:** Key Laboratory of Aquacultural Resources and Utilization, Ministry of Education, College of Fishery and Life Science, Shanghai Ocean University, Shanghai, China; Chinese Academy of Sciences, CHINA

## Abstract

DNA methylation is an essential epigenetic mechanism involved in multiple biological processes. However, the relationship between DNA methylation and cold acclimation remains poorly understood. In this study, Methylated DNA Immunoprecipitation Sequencing (MeDIP-seq) was performed to reveal a genome-wide methylation profile of zebrafish (Danio rerio) embryonic fibroblast cells (ZF4) and its variation under cold pressure. MeDIP-seq assay was conducted with ZF4 cells cultured at appropriate temperature of 28°C and at low temperature of 18°C for 5 (short-term) and 30 (long-term) days, respectively. Our data showed that DNA methylation level of whole genome increased after a short-term cold exposure and decreased after a long-term cold exposure. It is interesting that metabolism of folate pathway is significantly hypomethylated after short-term cold exposure, which is consistent with the increased DNA methylation level. 21% of methylation peaks were significantly altered after cold treatment. About 8% of altered DNA methylation peaks are located in promoter regions, while the majority of them are located in non-coding regions. Methylation of genes involved in multiple cold responsive biological processes were significantly affected, such as anti-oxidant system, apoptosis, development, chromatin modifying and immune system suggesting that those processes are responsive to cold stress through regulation of DNA methylation. Our data indicate the involvement of DNA methylation in cellular response to cold pressure, and put a new insight into the genome-wide epigenetic regulation under cold pressure.

## Introduction

DNA methylation of cytosine residues is a well-studied epigenetic mechanism implicated in multiple processes, such as genome function, gene transcription, imprinting and X-chromosome inactivation [1, 2]. Increasing number of studies show that DNA methylation plays an important role under various environmental pressures in some species [3–5]. In rice, widespread phosphate starvation-induced DNA methylation changes preferentially localize in transposable elements (TEs) close to highly induced genes [3]. In mouse liver and brain, highly variably DNA-methylated regions in environmental evolutionary adaptation were identified associated with development and morphogenesis [6]. In the plant, environmental stresses affect the epigenetic modifications and promote the activity of transposons for adaptation to the changing environment [7–10]. However, the role of DNA methylation in cold acclimation in fishes is still unclear.

Temperature is an important environmental factor that affects the life of fishes including development, sex determination, habit, and so on [11]. Fishes have been developing various adaptive changes for cold survival, including production of antifreeze glycoproteins (AFGP) [12], adaptive modification of enzyme protein structures [13], cold-efficient microtubule assembly [14], cold-adapted protein translocation [15], elevated mitochondrial densities [16], genomic expansion and gene loss [17, 18]. In addition to the mechanisms mentioned above, expression levels of many genes significantly change, contributing to adaptive changes of multiple processes including anti-oxidant system, apoptosis, development, immune system and so on [18]. In the transcriptomic study of cold acclimation in larval zebrafish, biological processes including RNA splicing, ribosome biogenesis and protein catabolic process were highly overrepresented [19, 20]. However the mechanisms underlying the regulation of those genes are still not clear.

Zebrafish is a major model system for studies of development, disease and other biological processes. DNA methylation profiling in zebrafish using whole genome bisulfite sequencing and MeDIP method has been reported [21–24], mainly investigating DNA methylation in gametes and early development. In another study, reduced representation bisulfite sequencing (RRBS) was used to profile DNA methylation patterns in the brain and liver of the zebrafish [25]. It is also known that DNA methylation dynamics occur during early zebrafish development [22]. However, the relationship between DNA methylation and environmental stress in zebrafish has not yet been reported. We wonder if DNA methylation variation takes place under cold stress and how. To address this question, ZF4 cells were used to investigate DNA methylation variation under cold pressure to eliminate tissue specific variations.

In this study, we investigated the role of DNA methylation in cold acclimation of zebrafish (Danio rerio) embryonic fibroblast ZF4 cells by genome-wide MeDIP-seq analysis. Our data suggested that DNA methylation is involved in the regulation of gene expression of essential biological processes needed for cold acclimation, such as anti-oxidant, apoptosis, development, chromatin modification. It is interesting that the global DNA methylation level increased at 5 days after cold treatment and decreased at 30 days after cold treatment, which is closely related to methylation level of genes involved in folate biosynthesis. Our data also suggested that DNA methylation of non-coding regions may play important roles in cold acclimation. The present work provides a novel insight into the-role of DNA methylation in cold acclimation in fishes.

## Materials and Methods

### Cell culture and treatment

ZF4 cell line was purchased from the American Type Culture Collection (ATCC, Cat No. CRL 2050). The cells were grown at 28°C, 5% CO_2_, in Dulbecco's modified Eagle's medium/F12 nutrient mix (SH30023.01B, Hyclone, Thermo Scientific) supplemented with 10% fetal bovine serum (10099141, Gibco, Life technologies), 1% penicillin-streptomycin-glutamine solution (SV30082.01, Hyclone, Thermo Scientific). For cold treatment, ZF4 cells were seeded at 40–50% confluence and the next day moved into an incubator at 18°C, 5% CO_2_, in the same medium for up to 30 days. The cells were split every 3 days, counted with a hemocytometer and reseeded at their initial density. All experiments were performed in triplicate. For cell viability assay, cells were prepared into a cell suspension and stained with 0.4% Trypan Blue solution (w/v) for 5 minutes, then counted with a hemocytometer.

### MeDIP-seq and statistical analysis

Genomic DNAs were isolated from ZF4 cells cultured at 28°C as control sample and at 18°C for 5 days (18°C /5d) and 30 days (18°C /30d), separately. Each condition has three biological replicates. Isolated genomic DNAs were sonicated and three biological replicates of sonicated genomic DNAs were pooled together for MeDIP-seq. The NEXTflex™ Methyl Sequencing 1 Kit (5118–01, Bioo Scientific) was used for end repair, adenylation, adapter ligation, methylated DNA enrichment and methylated DNA library construction according to the manufacturer’s instruction. Paired-end sequencing was performed with Illumina HiSeq 2000 sequencing system. Base calling was performed with Illumina Casava1.7 software. The FASTX-Toolkit program was used to filter off low quality sequences from raw sequencing data. High quality pair-end MeDIP-seq sequences were aligned with the zebrafish genome (Zv9/danRer7) downloaded from iGenome (http://support.illumina.com/sequencing/sequencing_software/genome.html) using Bowtie2 [26]. Methylated peaks were called using MACS software with default parameters. The three peak sets generated by MACS were merged using Bedtools for peaks with at least 1bp overlap to get a reference peak set (REF) for following analysis. Reads coverages were evaluated by read-count and reads per million (RPM) of REF, obtained by mapping Bowtie alignment results back to REF using Bedtools. DNA methylation pattern was revealed by hierarchical clustering of log2 (RPM+1) values of all detected methylation peaks of all samples. Reads coverage for 28°C, 18°C /5d, 18°C /30d were compared with each other with Fisher's Exact Test and FDR. Peaks with at least 2-fold change and with FDR< = 0.01 were selected as differentially methylated regions (DMR) for further analysis.

### Bisulfite sequencing PCR (BSP)

Genomic DNAs were bisulfite converted using EZ DNA Methylation-Gold kit (D5005, ZYMO) according to the instruction manual. The PCR primers were designed by MethPrimer (http://www.urogene.org/cgi-bin/methprimer/methprimer.cgi). Primers for *esrra* gene locus are BSP-esrra-F: 5'- AGGGTATGAGAGGAAAAATGT TTTA-3' and BSP-esrra-R: 5'-TTTTAAAAATACACCCCAAACAACT-3'. Primers for *cacng6a* gene locus are BSP-cacng6a-F: 5'-TGGGGTTTTAAATTTAAATGAGTTG-3' and BSP-cacng6a-R: 5'- AACAAACATCAATAACTCCACTAAC-3'. The PCR cycles were performed as following conditions: 94°C for 3 min, 37 cycles of 94°C for 30 s, 50°C for 30 s, 72°C for 30s, followed by 72°C for 5min. The PCR products were ligated into the pEASY-T5 Zero Cloning Vector (CT501, Transgen Biotech). Ten individual clones were sequenced in each sample.

### MeDIP-qPCR assay

MeDIP was performed using 1μg of DNA with EpiSeeker methylated DNA Immunoprecipitation (MeDIP) kit–DNA (ab117133, Abcam) according to the manufacturer’s instructions. Real-time PCR was carried out with MeDIP DNAs using Roche Light Cycler 480II System and Fast Start Universal SYBR Green Master (ROX) (04913949001, Roche Applied Science). Frequency of DNA methylation of immunoprecipitated DNA vs input DNA was calculated. Statistical analysis was performed using GraphPad Prism 5 software. The Student *t* test was used on measurements of enrichment frequency from 3 experimental replicates. Primers for MeDIP-qPCR are shown in S1 Table.

### Global DNA methylation level variation analysis of MeDIP-seq data

Global DNA methylation level analysis is based on pre-defined regions and RefSeq annotation. Pre-defined RefSeq genomic regions of upstream 3k, downstream 5k, 5’UTR, 3’UTR, exon and intron were downloaded from the UCSC table browser (https://genome.ucsc.edu/). Reads count along with RPKM for each region of each sample was calculated with Bedtools and an in-house R script. The RPKM value for each genomic region was compared for 18°C /5d vs 28°C, 18°C /30d vs 28°C, and 18°C /30d vs 18°C /5d using T test.

### Quantitation of global DNA methylation level

ZF4 cells were cultured at low temperature of 18°C up to 30 days. Genomic DNAs were isolated from ZF4 cells at time point of 1, 3, 5, 10, 15 and 30 days. Global DNA methylation levels were investigated by specifically measuring levels of 5-methylcytosine (5-mC) in an ELISA-like microplate-based format using MethylFlash Methylated DNA 5-mC Quantification Kit (P-1034-96, Epigenetics), following the manufacturer’s instruction. All experiments were performed in triplicate.

### Wilcoxon signed-rank test for KEGG analysis

Ensembl transcripts associated with peaks were linked with KEGG [27] ortholog IDs and pathway IDs using online KAAS annotation tool [28]. Peak sets related with each KEGG pathway were tested with Wilcoxon signed-rank test [29] to see if they are differentially distributed in the comparisons of 18°C /5d vs 28°C, 18°C /30d vs 28°C, and 18°C /30d vs 18°C /5d.

### GO enrichment analysis for differentially methylated regions (DMRs)

The Ensembl genome annotation was downloaded from the UCSC table browser (https://genome.ucsc.edu/). REF genomic type (e.g. promoter, intron…) annotation along with Ensembl transcript IDs were obtained by intersection of REF and Ensembl genome annotation using Bedtools [30]. REF was further linked with Ensembl gene IDs and GO annotation by Ensembl transcript to gene mapping and gene to GO mapping from Ensembl Biomart (http://www.ensembl.org/biomart/martview/7944b4c51c0cead1e48c21136c9c338f). Genes related with at least one DMR were used for GO enrichment analysis with in-house R scripts incorporating the R package of GO.db (GO.db: A set of annotation maps describing the entire Gene Ontology. R package version 3.1.2).

### Detection of the intracellular ROS (Reactive oxygen species) level

Intracellular ROS generation was measured using the fluorescent probe 2, 7-dichlorofluorescein diacetate (DCFH-DA) (D6883, Sigma-Aldrich). After cold treatment at 18°C for 0, 3, 6, 12, 24, 72 and 120 hours, ZF4 cells were washed twice with PBS, and then incubated with 20 μM DCFH-DA for 30 min at room temperature. The cells were washed three times with PBS, and collected for flow cytometer assay. ROS levels were expressed as median fluorescence intensity. All experiments were performed in triplicate and the student *t* test was performed using GraphPad Prism 5 software.

### Reverse transcription-qPCR analysis

Total RNA was isolated using TRlzol reagent (15596–026, Life Technologies). Reverse transcription (RT) was performed using 1μg of total RNA with Transcriptor First Strand cDNA synthesis kit (04379012001, Roche), according to the manufacturer’s instructions. PCR amplification was performed for 2 min at 50°C and 10 min at 95°C, followed by 40 cycles at 95°C for 10 s, and annealing at 60°C for 10 min. Relative mRNA level was analyzed by the comparative CT method. Data were normalized to β-actin. Statistical analysis was performed using GraphPad Prism 5 software. The Student *t* test was used on measurements of gene expression from cold treated and normal cultured samples from 3 experimental replicates. Primers for RT-qPCR analysis are shown in S2 Table.

### Chromatin immunoprecipitation (ChIP) analysis

ChIP was conducted according to a standard protocol [31]. In brief, cells were fixed in 1% formaldehyde for 6 min at room temperature. After lysis, samples were sonicated to a size range of 200 to 1,000 bps. Chromatin (150 to 200 μg) was immunoprecipitated with antibodies for H3K4me3 (ab8580, Abcam), H3K27me3 (17–622, Upstate) or rabbit IgG (15006, Sigma-Aldrich). A 10% aliquot was removed as an input fraction. Quantitative real time PCR was performed with ChIP DNA and input DNA. Data were normalized to IgG control. Relative quantitation was carried out by the comparative threshold cycle (CT) method. Statistical analysis was performed using GraphPad Prism 5 software. The Student *t* test was used on measurements of enrichment from cold treated and normal cultured samples from 3 experimental replicates. Primers for ChIP-qPCR are shown in S3 Table.

## Results

### Variation of the DNA methylation pattern of ZF4 cells under cold pressure

**Fig 1:**
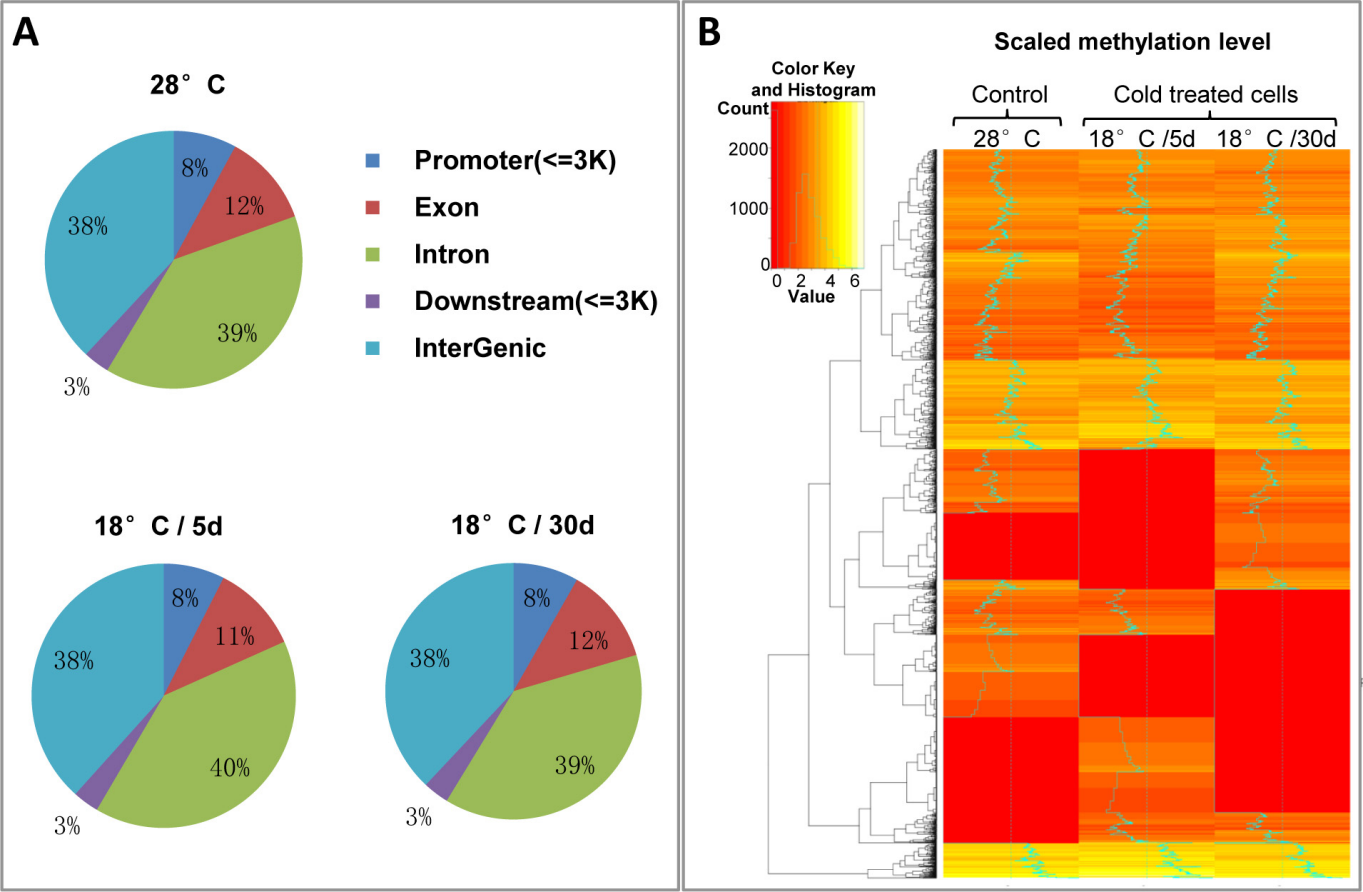
Variation of DNA methylation pattern of ZF4 cells under cold pressure. Genomic DNAs isolated from ZF4 cells cultured at 28°C, 18°C for 5 days and 30 days were subjected to MeDIP-seq analysis. **A**. Methylation peaks were distributed in promoter, exon, intron, downstream, intergenic regions as shown in the figure. **B**. Heat map of hierarchical clustering performed for all detected methylation peaks in all three samples shows variation of DNA methylation patterns under cold pressure. Each row represents a detected methylation peak.

To investigate the effect of cold pressure on the DNA methylation pattern of ZF4 cells, cells were moved from the appropriate culture condition of 28°C to a lower temperature of 18°C. Cells showed obvious growth arrest in the first 5 days under cold exposure, then cell growth accelerated and reached a stable state about 25 days after cold treatment, although still slower than cell growth at 28°C (S1A Fig). Then the short-term (18°C, 5 days) and long-term (18°C, 30 days) acclimated ZF4 cells were exposure to a lethal temperature of 10°C, the data indicated that both acclimated ZF4 cells showed significantly higher viability than non-acclimated ZF4 cells under the lethal cold pressure (S1B Fig). It suggested that ZF4 cells can develop cold acclimation under cold pressure. Genomic DNAs isolated from ZF4 cells cultured at 28°C, 18°C for 5 or 30 days were subjected to MeDIP-seq analysis, respectively. A range of 20,955,724 to 24,197,166 raw reads were generated from three samples after paired-end sequencing. More than 70% sequence reads were mapped to the zebrafish genome (Zv9/danRer7). Peak detection was performed using Model-based Analysis for ChIP-Seq (MACS) software [32]. Detected methylation peaks are distributed in the promoter, exon, intron, downstream, and intergenic regions by 8%, 12% or 11%, 39% or 40%, 3%, 38%, respectively (Fig 1A). Distribution of methylation peaks in different genomic regions shows similar pattern in those three samples. From our data, almost half number of 51% DNA methylation in the zebrafish genome is located in the gene body regions. CpG islands were predicted by Takai's CpG island searcher using GCC = 55 OE = 0.65 LENGTH = 500. About 7% of methylation peaks is located in CpG islands, which is consistent with the study of DNA methylation profiling in zebrafish brain [25]. DNA methylation pattern was generated by hierarchical clustering of log 2^(RPM+1)^ values of all detected methylation peaks from all three samples. As shown in Fig 1B, DNA methylation pattern is altered after cold treatment. Based on the cell response under cold stress, it is not surprising that DNA methylation patterns of 18°C /5d and 18°C /30d are different.

To verify the MeDIP-seq data, Bisulfite sequencing PCR (BSP) analysis was applied to investigate DNA methylation in two selected chromosomal loci. One locates at Chr.19: 10406775–10407187, ~2.3 kb upstream of *cacng6a* (voltage-dependent calcium channel, gamma subunit 6a) gene. Another locates at Chr.21: 25996283–25997258, ~9 kb upstream of *esrra* (estrogen-related receptor alpha) gene. It showed that percentage of methylated C in CpGI upstream of the two genes decreased after cold treatment, which is consistent with the MeDIP-seq data (S2 Fig). MeDIP-qPCR assay was also used to analyze seven gene loci: *gstk* (glutathione S-transferase kappa 1), *kdm4a* (lysine (K)-specific demethylase 4A), *aquaporin 1a*, *camk2a* (calcium/calmodulin-dependent protein kinase II alpha), *cacng6a*, *esrra* and an uncharacterized gene) (S3 Fig). All MeDIP-qPCR data are consistent with the MeDIP-seq data (S4 Table).

### DNA methylation level increased in short-term cold treatment

**Fig 2:**
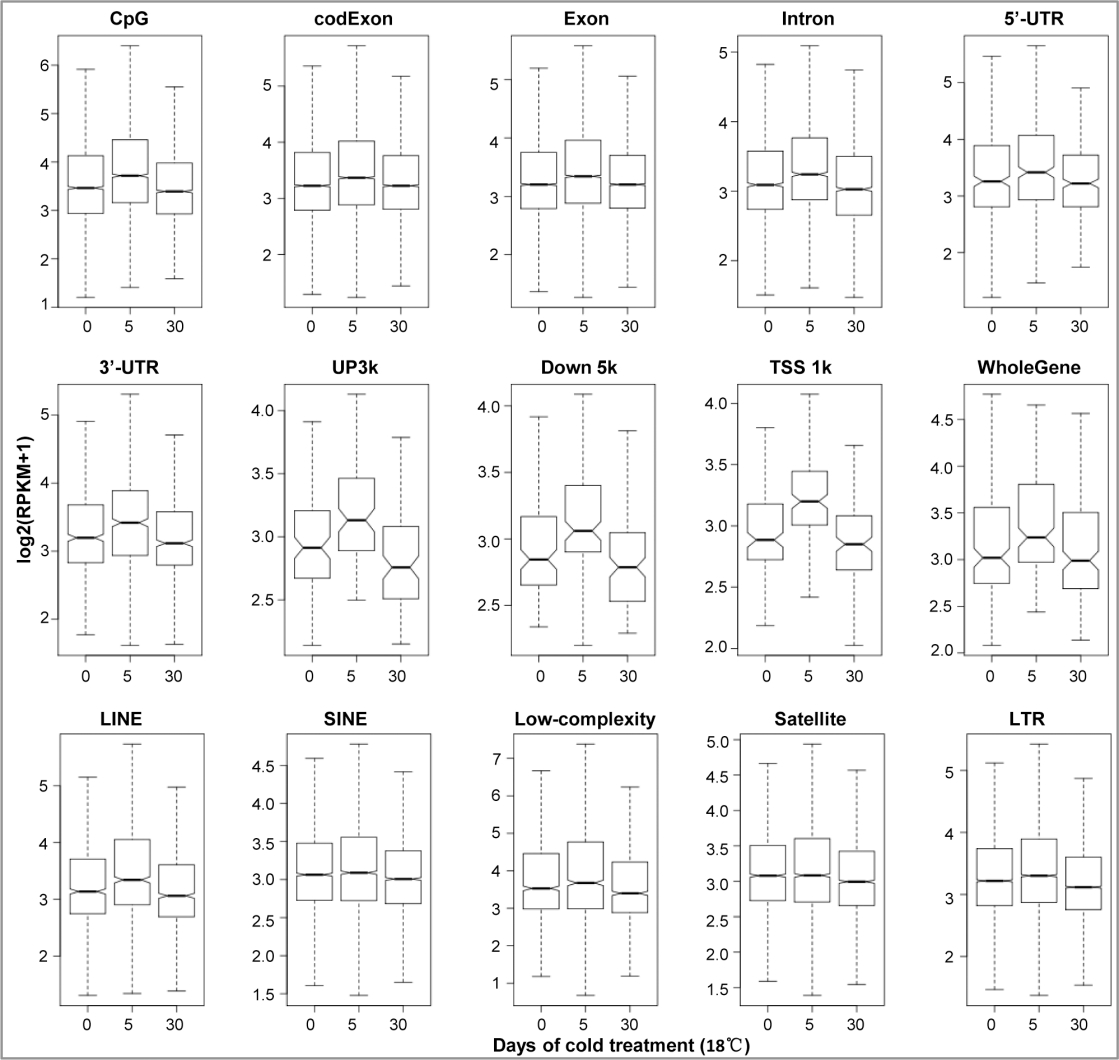
DNA methylation levels of different genomic regions under cold pressure. DNA methylation level of each genomic region from three samples (28°C, 18°C /5d, 18°C /30d) was shown as log2(RPKM+1). The RPKM value of each genomic region was compared as 18°C /5d vs 28°C, 18°C /30d vs 28°C and 18°C /30d vs 18°C /5d using T test. The corrected P values of FDR are shown in Table 1. CpG: CpG island; codexon: coding regions of exon; UP3k: regions within 3 kbs upstream gene; Down5k: regions within 5 kbs downstream gene; TSS 1k: regions within ±1 kb around transcription start site; LINE: long interspersed nuclear element; SINE: short interspersed nuclear element; Low complexity: low complexity DNA; Satellite: Satellite DNA; LTR: long terminal repeat.

For analysis of DNA methylation level variation, Bedtools [30] and an in-house R script were used for calculating Reads Per Kilobase per Million mapped reads (RPKM) for multiple types of genomic regions of each sample. The RPKM values were compared between different samples (18°C /5d vs 28°C, 18°C /30d vs 28°C and 18°C /30d vs 18°C /5d) using T test. Our data showed that DNA methylation levels of different genomic regions increased 5 days after cold treatment. And 30 days after cold treatment, DNA methylation levels in upstream, downstream, intron and repeat sequences became lower than those of control cells (Fig 2). The corrected P values of multiple testing corrections (False Discovering Rate, FDR) of the comparisons are shown in Table 1. The variation of DNA methylation level was also confirmed by quantitation assay of global genomic DNA methylation with Methyl Flash Methylated DNA 5-mC Quantification Kit. Our data showed that DNA methylation level increased after cold treatment and reached a peak on the 5th day then decreased to a level slightly lower than that of control cells 30 days after cold treatment (S4A Fig). By contrast, the global DNA methylation level of ZF4 cells remained unchanged after 5 and 30 days at 28°C (S4B Fig), indicating ZF4 cells were stable and maintained constant DNA methylation level in this study. Above data indicated that genome-wide DNA methylation variation occurred in response to cold stress.

**Table 1:** FDRs of the methylation level comparisons from three different samples (28°C, 18°C /5d and 18°C /30d).

Genomic regions	18°C /5d vs 28°C	18°C /30d vs 28°C	18°C /30d vs 18°C /5d
CpG	7.790230e-36	0.000000e+00	0.000000e+00
Codexon	1.002201e-25	0.000000e+00	0.000000e+00
Exon	1.561321e-25	0.000000e+00	0.000000e+00
Intron	1.555422e-10	0.000000e+00	0.000000e+00
UTR5	1.810113e-02	5.875863e-182	6.651023e-189
UTR3	3.028749e-06	0.000000e+00	0.000000e+00
UP 3K	4.952519e-03	6.974608e-65	1.292772e-66
Down 5K	3.582671e-04	1.817584e-87	2.162776e-86
TSS (1K)	8.000152e-05	4.503675e-104	8.264075e-109
Wholegene	9.338125e-04	4.102350e-109	7.909553e-114
LINE	3.235575e-08	1.211646e-02	1.150671e-15
SINE	7.867506e-02	8.986845e-05	2.826310e-08
Low-complexity	1.361181e-03	1.106698e-06	7.460555e-15
Satellite	6.856858e-02	8.638236e-06	1.245566e-09
LTR	1.243673e-11	9.470906e-17	9.124971e-50

### Folate biosynthesis pathway was significantly hypomethylated after short-term cold exposure

**Fig 3:**
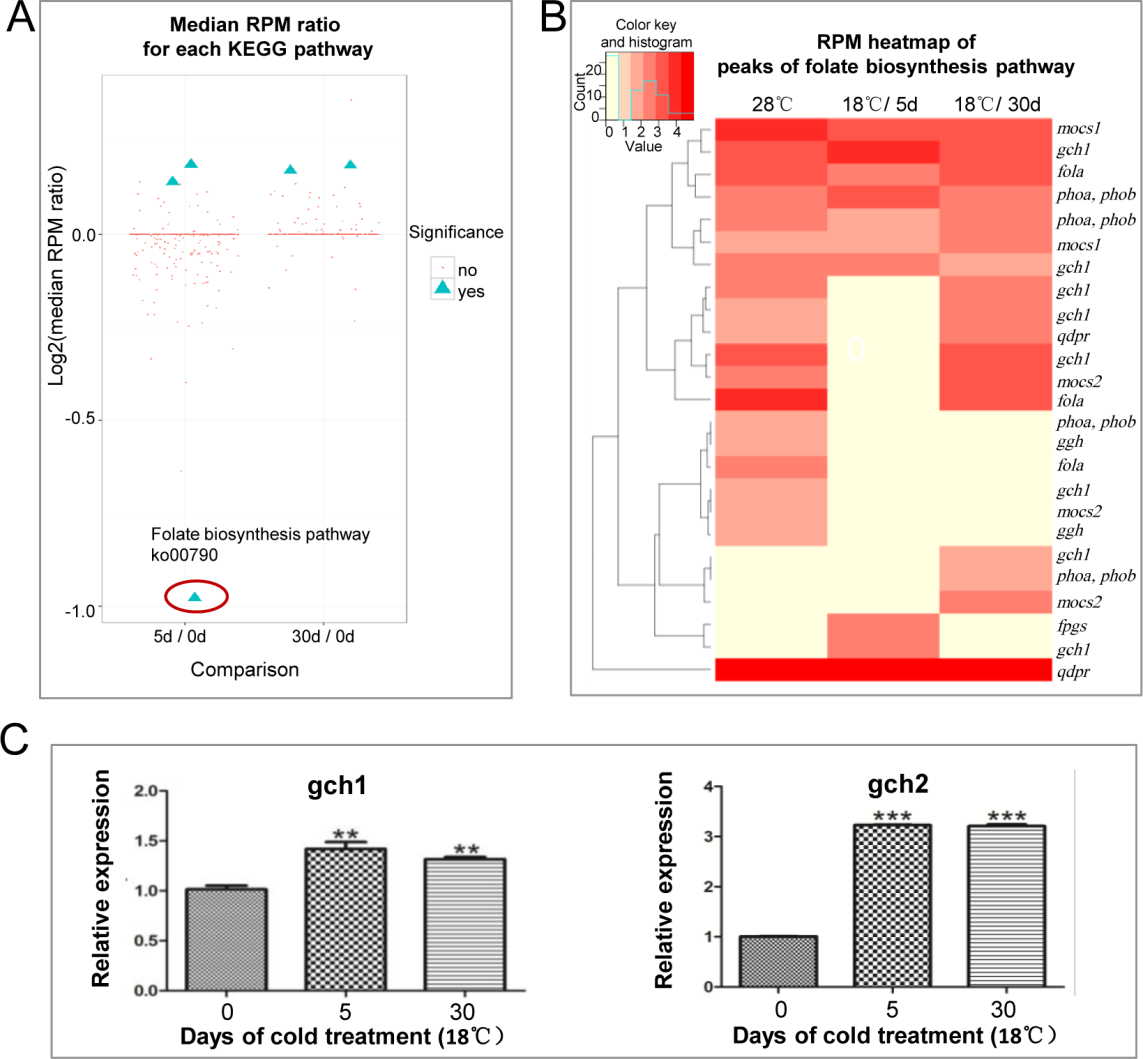
Folate biosynthesis pathway (ko00790) was differentially distributed in 5- day cold treated cells. A. Differentially distributed KEGG pathways in cold treated cells compared with control cells. Wilcoxon signed-rank test was applied to identify KEGG pathways of which related peak-RPM values are differentially distributed in any of the two sample pairs (5d/0d: 18°C /5d vs 28°C, 30d/0d: 18°C /30d vs 28°C). We identified 5 KEGG pathways as differentially distributed that meet the criteria of P value < = 0.05 and Median RPM Ratio > = 1.1 or < = 1/1.1 (marked by blue solid triangles). Red dots present non-significantly changed KEGG. Folate biosynthesis pathway (ko00790) is marked by red circle. B. RPM heatmap of methylation peaks of folate biosynthesis pathway. Peak-RPM of folate biosynthesis pathway in three different samples (28°C, 18°C /5d, 18°C /30d) is shown by the density of the red color. C. RT-qPCR analysis of *gch1* and *gch2* genes. Relative expression was carried out by the comparative threshold cycle (CT) method. Statistical analysis was performed using GraphPad Prism 5 software. The Student *t* test was used on measurements from18°C /5d, 18°C /30d and 28°C samples from 3 experimental replicates.

Wilcoxon signed-rank test [33] was applied to identify Kyoto Encyclopedia of Genes and Genomes (KEGG) pathways of which RPKM values are differentially distributed in any of the two sample pairs (18°C /5d vs 28°C, 18°C /30d vs 28°C). 5 KEGG pathways were identified as differentially distributed that meet the criteria of P value < = 0.05 and Median RPM Ratio > = 1.1 or < = 1/1.1(Table 2, Fig 3A). They are ko00524 (Butirosin and neomycin biosynthesis), ko00601 (Glycosphingolipid biosynthesis—lacto and neolacto series), ko00790 (Folate biosynthesis), ko00140 (Steroid hormone biosynthesis) and ko00591 (Linoleic acid metabolism). It’s interesting that folate biosynthesis pathway was remarkably hypomethylated in 5-day cold treated cells. Genes involved in folate biosynthesis pathway were detected hypomethylated (Fig 3B), including GTP cyclohydrolase I (*gch*), GTP cyclohydrolase 2 (*gch2*), dihydropteridine reductase (*qdpr*), *mocs1* (*moaA*, molybdenum cofactor biosynthesis protein), *mocs2* (*moaE*, molybdopterin synthase catalytic subunit), alkaline phosphatase (*phoA*, *phoB*), folylpolyglutamate synthase (*fpgs*) and dihydrofolate reductase (*folA*). Expression of *gch1* and *gch2* were detected up-regulated after cold treatment, consistent with decreased DNA methylation of those two genes (Fig 3C). Folate is well known as an important active methyl-group donor in organisms, and the amount of the folate directly affects methylation reaction [34]. Considering that global DNA methylation level was up-regulated at the same time point, there might be a direct correlation between DNA methylation and folate biosynthesis activity under cold pressure.

**Table 2:** Wilcoxon signed-rank test of KEGG in cold treatment cells (Median RPM Ratio > = 1.1 or < = 1/1.1, median_test P value < = 0.05).

**Ko ID**	**Ko_median 18°C/5d vs 28°C**	**P-value**	**Ko description**
ko00524	1.103	0.04686926	Butirosin and neomycin biosynthesis
ko00601	1.1395	0.01222566	Glycosphingolipid biosynthesis—lacto and neolacto series
ko00790	0.508	0.02300543	Folate biosynthesis
**Ko ID**	**Ko_median 18°C /30d vs 28°C**	**P-value**	**Ko description**
ko00140	1.1265	0.00831028	Steroid hormone biosynthesis
ko00591	1.137	0.03674186	Linoleic acid metabolism

### Gene Ontology (GO) enrichment analysis of differentially methylated regions (DMRs)

To explore biological significance of DNA methylation variation under cold pressure, we used Gene Ontology analysis to examine the functional enrichments of genes with cold related differential methylation. For identification of differentially methylated regions (DMRs), reads coverage for 28°C, 18°C /5d and 18°C /30d were compared with each other using Fisher's Exact Test and FDR. Peaks with at least 2-fold change and with FDR< = 0.01 were selected as DMRs. DMRs are distributed in different chromosomal regions, including promoter, exon, intron and intergenic regions. There are 12,590 methylation peaks altered in both 5-day and 30-day cold treated cells, compared with control cells, and we named these altered regions as cold-related methylation regions (CRMs). It was reported that 87% of active promoters are located within 2.5k upstream of transcription start site (TSS) [35]. Regions located within 3 kb upstream of TSS are considered as promoter regions [36, 37]. About 8% of CRMs are located in promoter regions. The majority of CRMs are located in intergenic regions.

**Fig 4:**
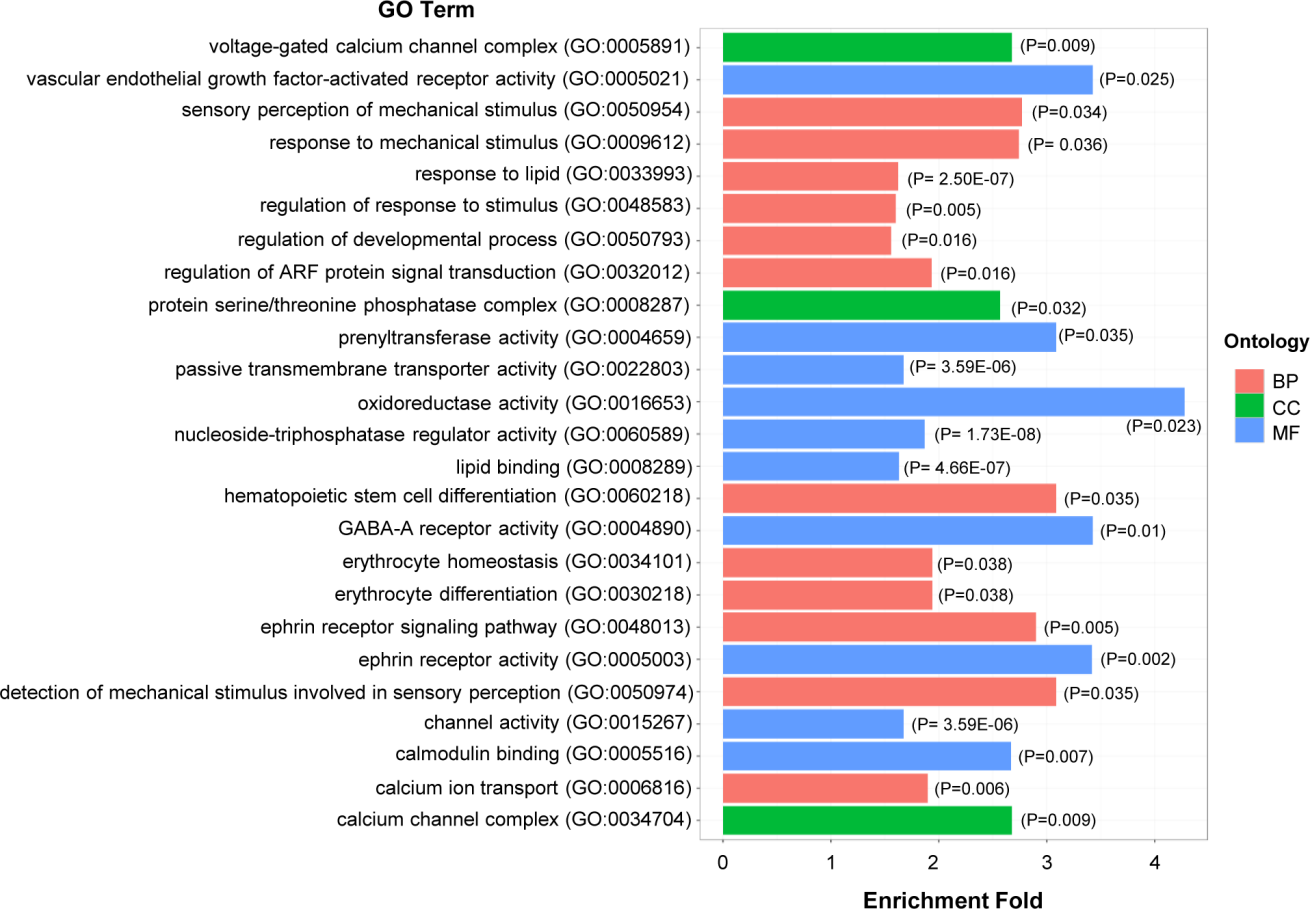
Enriched GO categories under cold pressure. GO enrichment analysis was performed in both 5-day and 30-day cold treated cells (cultured at 18°C) compared with control cells (cultured at 28°C). Genes related with DMRs were subjected to the analysis. GOs with ratio > = 1.5 and P value < = 0.05 are shown in the figure. P values for enrichment of GO categories are shown in the figure.

GO enrichment analysis was performed in both 5-day and 30-day cold treated cells compared with control cells. Genes related with at least one DMR are used for the analysis. Those with P value < = 0.05 and ratio > = 1.3 were considered as significantly enriched GO (S5 Table). Totally 203 GO categories were identified enriched after cold treatment. The enriched GO categories with P value < = 0.05 and ratio > = 1.5 are shown in Fig 4. Enriched GOs conduct biological processes such as anti-oxidant activity, regulation of response to stimulus, lipid metabolism, nucleoside-triphosphatase regulator activity, calmodulin binding, calcium ion transport, regulation of developmental process and so on. It suggested that these biological processes might be involved in cellular response to cold pressure though DNA methylation regulation.

### Altered DNA methylation in promoter regions is associated with gene expression in cold acclimation

**Fig 5:**
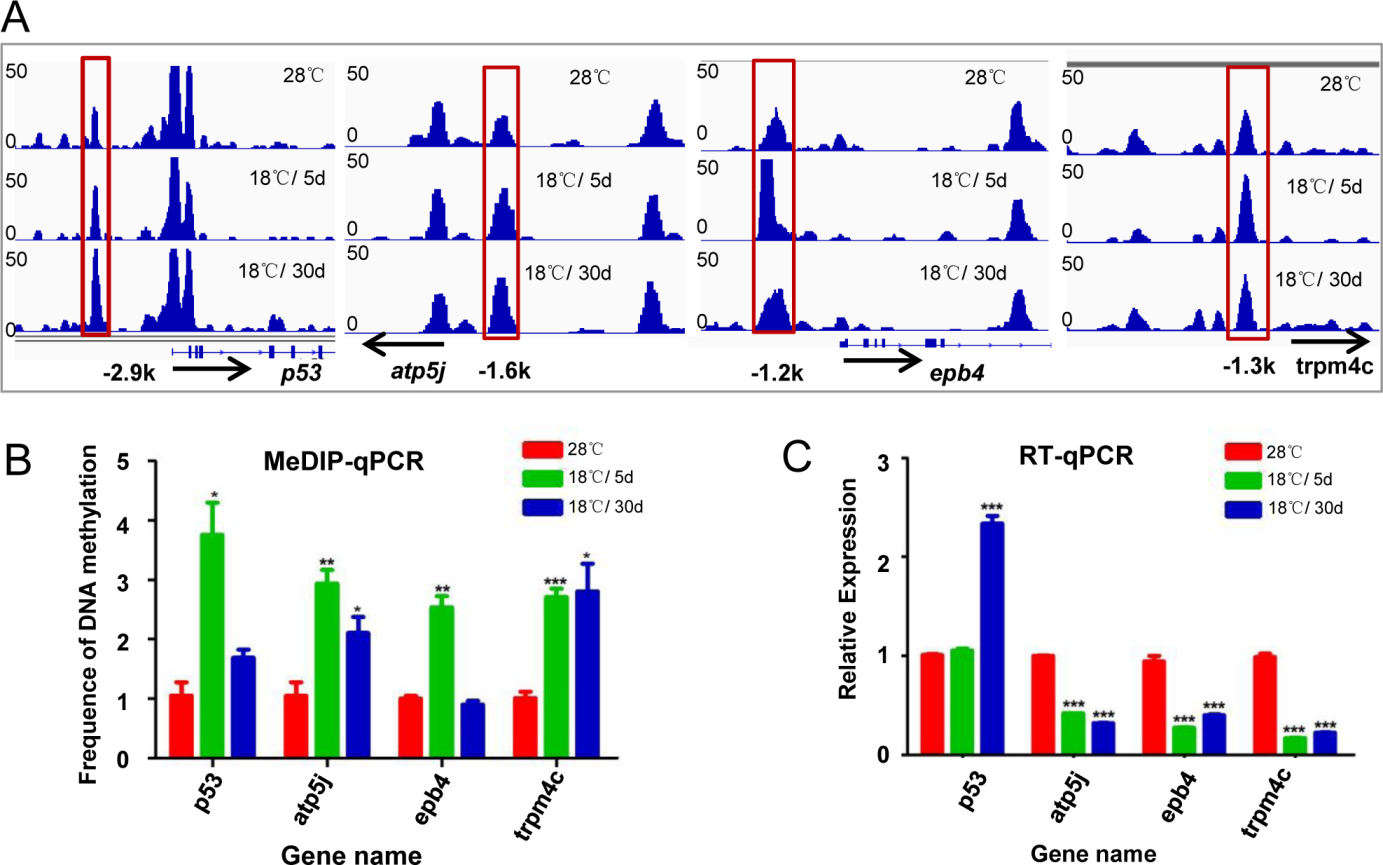
DNA methylation in promoter regions of cold-related genes and expression analysis. A. IGV images created from MeDIP-seq data for *p53*, *atp5j*, *epb4* and *trpm4c* gene loci. Black arrows show the transcriptional direction of genes. Red boxes show differentially methylated peaks upstream of genes. B. MeDIP-qPCR analysis of *p53*, *atp5j*, *epb4* and *trpm4c*. Frequency of DNA methylation was calculated by the comparative threshold cycle (CT) method. C. RT-qPCR analysis of *p53*, *atp5j*, *epb4* and *trpm4c*. Relative quantitation was carried out by the comparative threshold cycle (CT) method. Statistical analysis was performed using GraphPad Prism 5 software. The student *t* test was used on measurements from 18°C /5d, 18°C /30d and 28°C samples from 3 experimental replicates. Error bars represent standard deviations (SD) (n = 3). Data are normalized to β-actin. An asterisk represents significant difference compared to 28°C sample (P<0.05). **: P<0.01, ***: P<0.001.

It is well documented that DNA methylation level at promoter regions is highly related to gene expression [38, 39]. We pursued how DNA methylation variation affects gene expression under cold pressure. As mentioned above, only 8% of CRMs locate in gene promoter regions. Totally 1,024 genes were detected with altered DNA methylation in promoter regions after cold treatment (S4 Table). These genes are mostly involved in biological processes previously reported related to cold adaptation [18], including apoptosis, antioxidant system, chromatin modifying system, transportation, energy metabolism and immune system. Expression of *p53*, *atp5j* (*ATP synthase*), *epb41b* (*erythrocyte membrane protein band 4*.*1b*) and *trpm4* (transient receptor potential cation channel, subfamily M, member 4) genes involved in apoptosis, energy metabolism, development and transportation were analyzed. MeDIP-qPCR data of these genes showed the same trend with MeDIP-seq data (Fig 5A and 5B). By contrast, MeDIP-qPCR showed DNA methylation levels of these genes were stable in ZF4 cells cultured at 28°C for 5 or 30 days (S5A Fig). RT-qPCR data showed that mRNA levels of *atp5j*, *epb41b* and *trpm4* were changed and negatively related to DNA methylation variation (Fig 5C). By contrast, mRNA levels of these genes showed nonsignificant changes in ZF4 cells cultured at 28°C for 5 or 30 days (S5B Fig). However, mRNA level of *p53* gene is not negatively related to DNA methylation variation at the promoter region.

**Fig 6:**
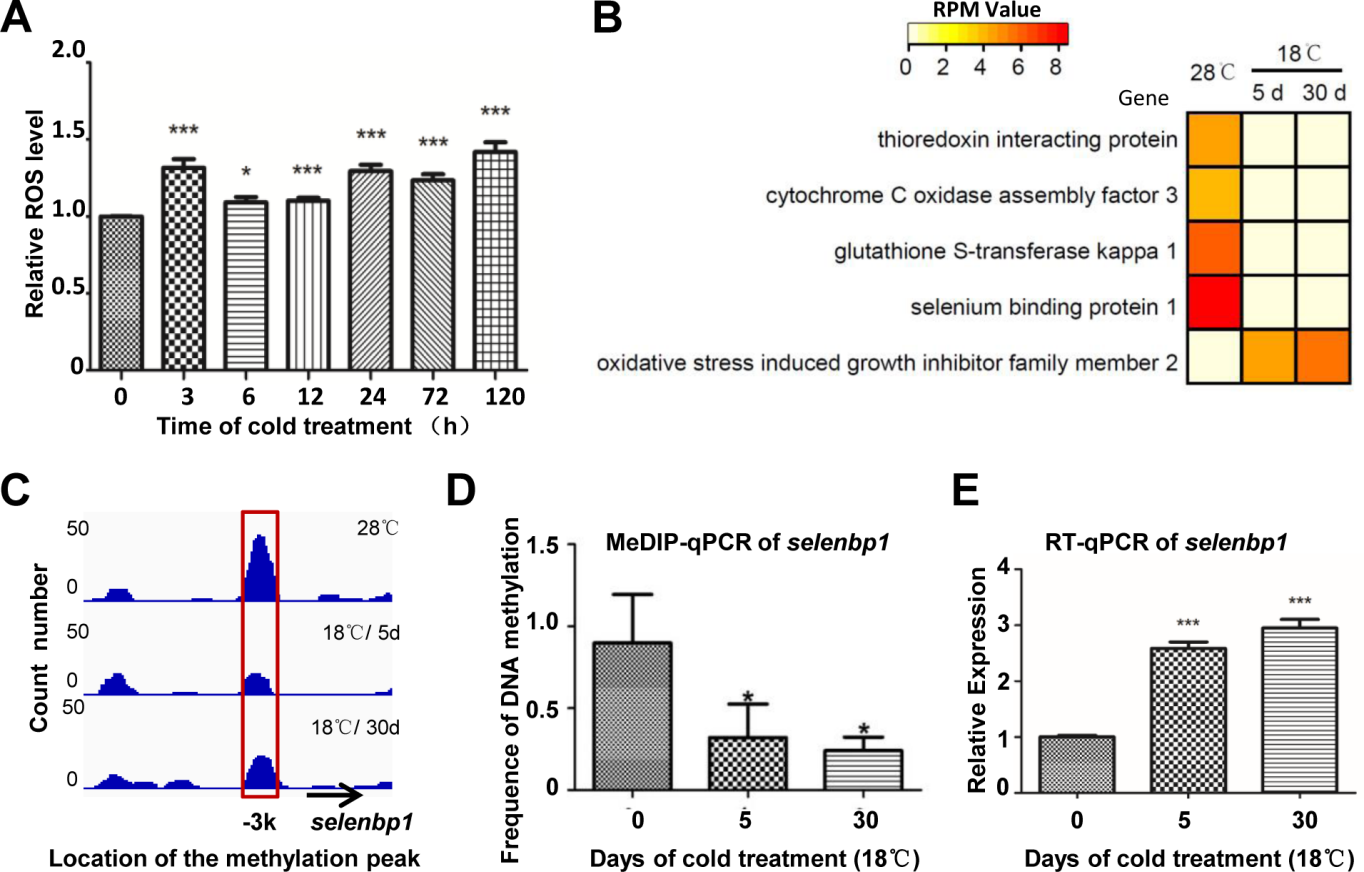
Anti-ROS genes were hypomethylated under cold pressure. A. Cold stress induced ROS production in ZF4 cells. ZF4 cells were treated with cold stress at 18°C for 3 h, 6 h, 12 h, 1 d, 3 d and 5 d. The DCF positive cells were measured by flow cytometric analysis. Error bars represent standard deviations (SD) (n = 3). An asterisk represents significant difference of ROS level compared to 28°C sample (P<0.05). **: P<0.01, ***: P<0.001. B. Heatmap of RPM values of DNA methylation at promoter regions of anti-ROS genes. C. IGV image created from MeDIP-seq data for *selenbp1* gene. Black arrow shows the transcriptional direction of the gene. Red box shows differentially methylated peaks upstream of the gene. D. MeDIP-qPCR analysis of *selenbp1* gene. Frequency of DNA methylation was calculated by the comparative threshold cycle (CT) method. E. RT-qPCR analysis of *selenbp1* gene. Relative quantitation was carried out by the comparative threshold cycle (CT) method. Error bars represent standard deviations (SD) (n = 3). Data are normalized to β-actin. An asterisk represents significant difference compared to 28°C sample (P<0.05). **: P<0.01, ***: P<0.001.

It is reported that anti–ROS (Reactive oxygen species) is a major protective process in cold adaptation in Antarctic fishes [18]. In our study, ROS level increased in cold treated ZF4 cells (Fig 6A). What’s interesting is DNA methylation changes were detected in promoter regions of a number of genes involved in anti-oxidant system, including *gstk* (glutathione S-transferase kappa 1), *selenbp1* (selenium binding protein 1), *coa3* (cytochrome C oxidase assembly factor 3), *txnip* (thioredoxin interacting protein), *osgin2* (oxidative stress induced growth inhibitor family member 2) (Fig 6B). Except for *osgin2* gene, above mentioned genes were hypomethylated in their promoter regions after cold treatment. As a sensor of ROS, *selenbp1* gene was selected for detailed analysis. DNA methylation at ~3kb upstream of *selenbp1* gene remarkably decreased under cold stress (Fig 6C and 6D), but remained unchanged at 28°C within 30 days (S5A Fig). RT-qPCR analysis showed that expression of the gene increased, consistent with remarkable decline of DNA methylation at the promoter region (Fig 6E). Our data suggested anti-ROS activity increased to protect cells from cold pressure through DNA methylation regulation.

**Fig 7:**
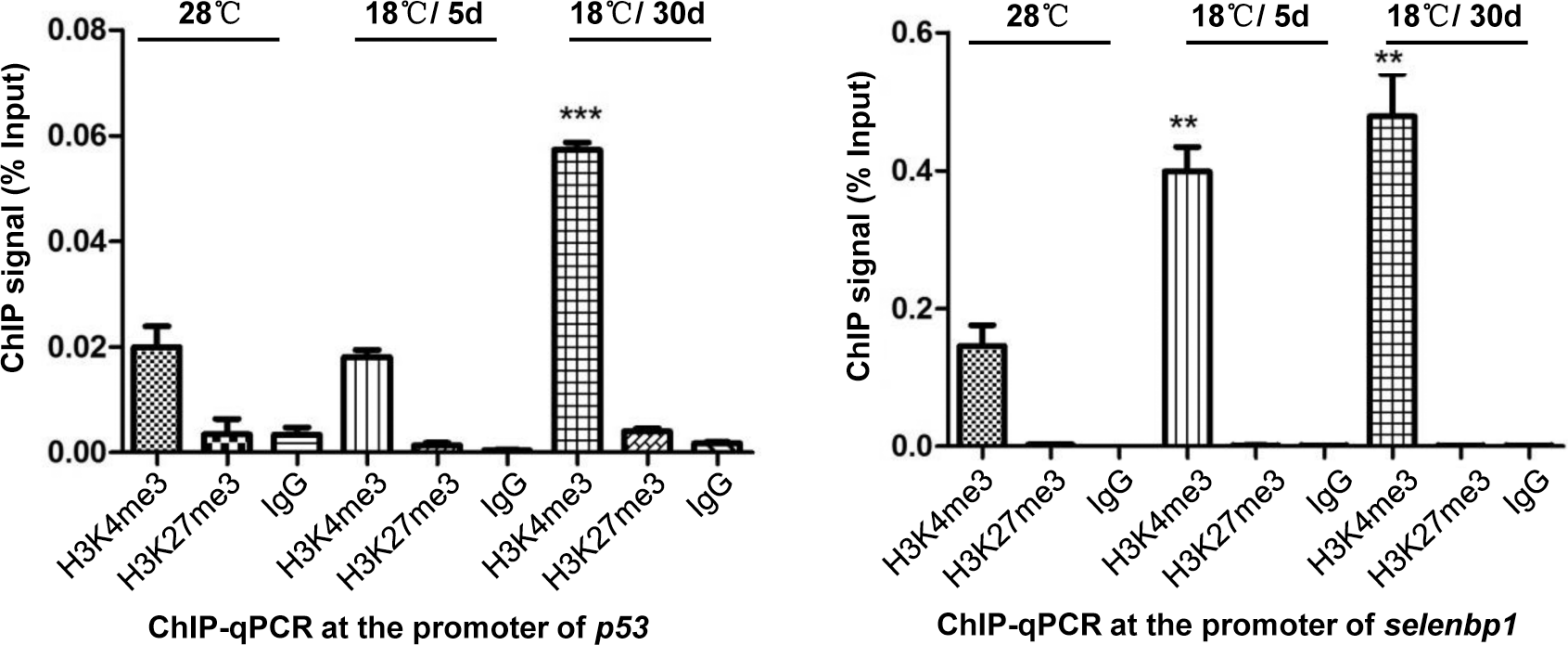
Enrichment of H3K4me3 and H3K27me3 at promoter regions of p53 and selenbp1 gene. ChIP assays were performed in 28°C, 18°C /5d and 18°C /30d samples with H3K4me3, H3K27me3 and IgG antibodies. Primers specific for the promoters of *p53* (A) and *selenbp1* (B) were used for quantitative PCR of ChIP DNAs. Relative quantitation was carried out by the comparative threshold cycle (CT) method. Statistical analysis was performed using GraphPad Prism 5 software. The Student *t* test was used on measurements of enrichment from 18°C /5d, 18°C /30d and 28°C samples from 3 replicates. Error bars represent standard deviations (SD) (n = 3). An asterisk represents significant difference of enrichment compared to 28°C sample (P<0.05). **: P<0.01, ***: P<0.001.

Epigenetic regulation of gene expression involves multiple processes including covalent modifications of DNA, histones and chromatin structure. To investigate the possibility that other epigenetic mechanisms compromise the action of DNA methylation in the regulation of specific genes mentioned above, we did chromatin immunoprecipitation assay (ChIP-qPCR) in promoter regions of *p53* and *selenbp1* gene with positive (H3K4me3) and repressive (H3K27me3) histone marks. Enrichment of histone modifications at promoter regions of both genes also changed after cold treatment (Fig 7). And the state of histone modification marks is more consistent with gene expression. Our data suggested that multiple epigenetic mechanisms are involved in cold induced gene expression alteration.

## Discussion

DNA methylation is a major epigenetic mechanism that regulates gene expression. Increasing number of studies suggest environmentally-induced DNA methylation changes contribute to adaptive phenotypic variation, which influences survival or/and provides a substrate for selection [40]. Although relationship between low temperature and DNA methylation in plants has been reported [41–43], the roles of DNA methylation and other epigenetic mechanisms in cold acclimation are still unclear, despite of increasing number of studies to understand the mechanisms of cold acclimation in fishes.

Zebrafish is a useful model organism in developmental biology and genetic studies. It is reported that zebrafish showed decreased metabolic rate when experimentally acclimated at 18°C for 28 days [44], and CDC48 mRNA level increased significantly in zebrafish cells after 25 days at 15°C to promote cell proliferation [45]. Here we cultured zebrafish ZF4 cells at 18°C for 5 and 30 days for short and long-term cold acclimation and investigated the role of DNA methylation in this process.

Using genome-wide MeDIP-seq analysis, we obtained the DNA methylation profile of ZF4 cells and detected its variation in short-term (18°C /5d) and long-term (18°C /30d) cold treated cells. We found that DNA methylation profile significantly changed both 5 days and 30 days after cold exposure. Expression levels of many genes of multiple well studied cold responsive biological processes showed negative correlation with DNA methylation in the promoter regions, suggesting DNA methylation is closely involved in the regulation of those genes under cold pressure.

Most cold responsive pathways have been accepted essential for cold survival and cold adaptation. For example, enhanced anti-ROS activity has been reported as the principal mechanism for cold adaptation via protecting cells from cold induced ROS [18]. In this study, genes of anti-ROS system were detected hypomethylated under cold pressure, which at least partly account for the increased anti-ROS activity required for cold acclimation. Surprisingly, consistent with the report that cold exposure down-regulates zebrafish hematopoiesis [46], DNA methylation in the promoter region of *erythrocyte membrane protein band 4*.*1b* (*epb4*.*1b*) changed significantly after cold treatment, and mRNA level of *epb4*.*1b* was down-regulated (Fig 5C), indicating this change is not unique in blood cells. Genes involved in chromatin modifying were affected significantly in cold treated cells, such as *lysine (K)-specific demethylase 4A* (*kdm4a*), *methyltransferase like 6* (*mettl 6*), *histidine decarboxylase* (*dhc*), suggesting the involvement of additional epigenetic mechanisms (S4 Table). Those data indicate that DNA methylation in promoter regions may play important roles in cold acclimation of fish cells.

Global genomic DNA methylation level increased in short-term cold treated cells and then decreased to a slightly lower level in long-term cold acclimated cells, although the trends of DNA methylation of many cold responsive genes mentioned above are same at both time points. It's interesting that genes of folate biosynthesis pathway are significantly hypomethylated after short-term cold exposure in ZF4 cells, so that change of folate biosynthesis activity shows the same trend with global genomic DNA methylation level. Considering that folate is a key active methyl-group donor for DNA methylation [34, 47, 48], the correlation between folate biosynthesis, DNA methylation, and short-term cold response will be explored. Folate not only affects DNA methylation reaction, but also may play roles in cold adaptation via other mechanisms. Anti-oxidative effects of folic acid have been reported [49, 50], and folate can alleviate cold-induced elevation of homocysteine and lipid peroxidation [51], suggesting another role of folate biosynthesis in cold response of fish cells.

The majority of CRMs are located in non-coding regions, indicating DNA methylation in non-coding regions might play more important roles in gene regulation under cold pressure. It is widely accepted that many non-coding DNAs, including enhancer, repressor, insulator regulatory elements and small RNAs genes, play important roles in gene regulation involving multiple processes. It is also reported recently that DNA methylation in enhancer regions [52], insulator regions [53], and non-coding RNAs gene regions [54] is related with gene expression regulation, so DNA methylation of those non-coding regions might also play roles during cold acclimation in fish cells. In our study, DNA methylation levels in repeat sequences decreased in long-term cold acclimation cells, which could contribute to understanding of the extensive duplication of LINEs (Long interspersed nuclear elements) in the Antarctic species under constant cold pressure [18].

DNA methylation is not the only epigenetic mechanism involved in cold acclimation in fish cells. Our data showed that altered enrichment of two histone modifications in promoter regions of two cold responsive genes (*p53* and *selenbp1*) affect gene expression, indicating other epigenetic mechanisms might play roles in cold acclimation of fish cells. Variation of DNA methylation and expression of chromatin modifying enzymes under cold pressure in this study also indicates the involvement of multiple epigenetic mechanisms. It is interesting to investigate how other epigenetic mechanisms, such as histone modifications and chromatin structures, are involved in cold acclimation.

Our study indicated fish cells possess the cold tolerance ability under low temperature and DNA methylation is extensively involved in this acclimation process.

## Supporting Information

S1 Fig Growth and viability assay of ZF4 cells under cold pressure. A. ZF4 cells were cultured at 18°C for up to 30 days. Long-term growth assay were performed for 0, 5, 10, 15, 20, 25 and 30 days. B. ZF4 cells were cultured at 18°C for 5 or 30 days for short-term or long-term acclimation, then acclimated and non-acclimated (28°C) ZF4 cells were exposure to 10°C for 3 days, the cell viability was measured by Trypan blue exclusion test.
(PDF)

S2 Fig Bisulfite sequencing PCR (BSP) assay for esrra and cacng6a gene loci. Genomic DNAs from different samples were bisulfite converted and amplified with specific primers for *esrra* (A) and *cacng6a* loci (B). Ten individual clones were sequenced for each sample. IGV images created from MeDIP-seq data for esrra locus are shown in Fig A. Black dots present methylated CpG and circles present un-methylated CpG. Percentage of relative methylated CpGs is shown in the figure.
(PDF)

S3 Fig MeDIP-qPCR assay of selected gene loci. Isolated genomic DNAs were subjected to MeDIP assay. Frequency of DNA methylation of immunoprecipitated DNA was calculated by the comparative threshold cycle (CT) method. Statistical analysis was performed using GraphPad Prism 5 software. The Student t test was used on measurements from 28°C, 18°C /5d and 18°C /30d samples from 3 experimental replicates. An asterisk represents significant difference compared to 28°C sample (P<0.05). **: P<0.01, ***: P<0.001.
(PDF)

S4 Fig Quantitation assay of global genomic DNA methylation of ZF4 cells under cold pressure. Genomic DNAs were isolated from cells cultured at 18°C (A) or 28°C (B) for indicated times. Quantitation assay of global genomic DNA methylation was performed with MethylFlash Methylated DNA 5-mC Quantification Kit. Percentage of DNA methylation was calculated according to the protocol. Error bars represent standard deviations (SD) (n = 3). An asterisk represents significant difference of signal compared to 28°C sample (P<0.05). **: P<0.01. ***: P<0.001.
(PDF)

S5 Fig DNA methylation levels in promoter regions and mRNA levels of cold-related genes in ZF4 cells under normal culture condition. ZF4 cells were cultured at 28°C for 5 or 30 days, then MeDIP-qPCR (A) and RT-qPCR (B) were performed to detect DNA methylation levels in promoter regions and mRNA levels of indicated genes. Frequency of DNA methylation and relative expression were carried out by the comparative threshold cycle (CT) method. Statistical analysis was performed using GraphPad Prism 5 software. The student t test was used on measurements from 3 experimental replicates. Error bars represent standard deviations (SD) (n = 3). An asterisk represents significant difference compared to 28°C sample (P<0.05). **: P<0.01, ***: P<0.001.
(PDF)

S1 Table Primers for MeDIP-qPCR analysis. (XLSX)

S2 Table Primers for RT-qPCR analysis. (XLSX)

S3 Table Primers for ChIP-qPCR analysis. (XLSX)

S4 Table DNA methylation variation in promoter regions after cold treatment (Fold > = 2 or < = 0.5, FDR< = 0.01). (XLSX)

S5 Table Enriched GO categories of DMRs after cold treatment (Fold >1.3, P<0.05, FDR<0.2). (XLSX)

## Abbreviations

MeDIP: Methylated DNA immunoprecipitation
CGI: CpG island
ChIP: Chromatin immunoprecipitation
GO: Gene ontology
TSS: Transcription start site
RPM: Reads per million
RPKM: Reads Per Kilobase per Million mapped reads
BSP: Bisulfite sequencing PCR
DMR: Differentially methylated regions
ROS: Reactive oxygen species
