# Supplementary figures and images for: Variation of DNA Methylome of Zebrafish Cells under Cold Pressure
Source: PLoS One. 2016 Aug 5;11(8):e0160358. doi: 10.1371/journal.pone.0160358 (PMC4975392; doi:10.1371/journal.pone.0160358)

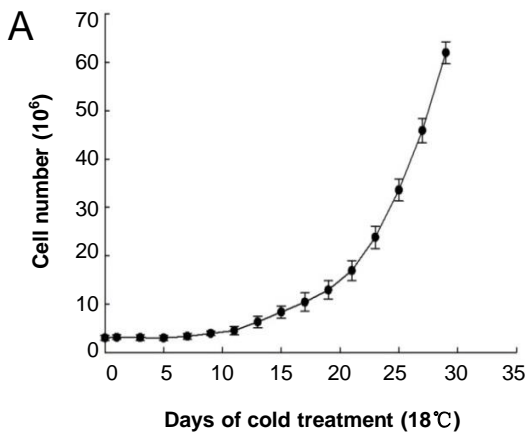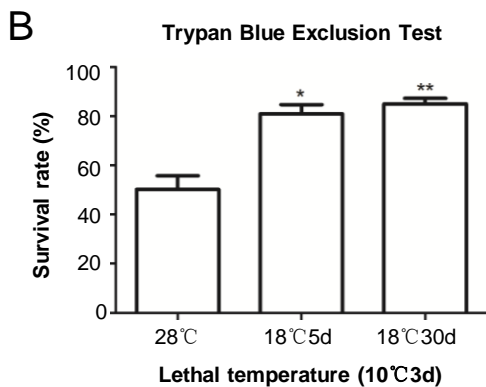

Supplement: S1 Fig — A. ZF4 cells were cultured at 18°C for up to 30 days. Long-term growth assay were performed for 0, 5, 10, 15, 20, 25 and 30 days. B. ZF4 cells were cultured at 18°C for 5 or 30 days for short-term or long-term acclimation, then acclimated and non-acclimated (28°C) ZF4 cells were exposure to 10°C for 3 days, the cell viability was measured by Trypan blue exclusion test. (PDF) [file pone.0160358.s001.pdf]

## MeDIP-qPCR

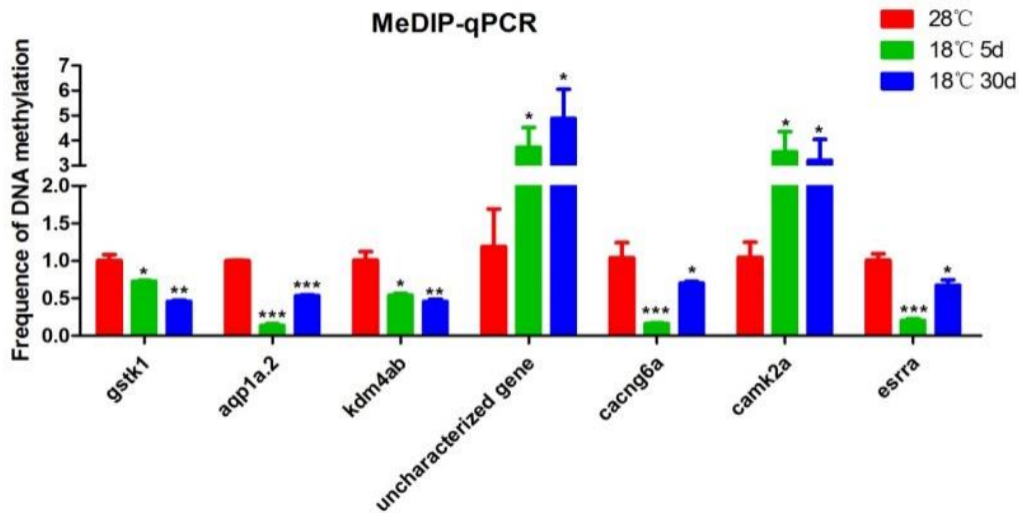

Supplement: S3 Fig — Isolated genomic DNAs were subjected to MeDIP assay. Frequency of DNA methylation of immunoprecipitated DNA was calculated by the comparative threshold cycle (CT) method. Statistical analysis was performed using GraphPad Prism 5 software. The Student t test was used on measurements from 28°C, 18°C /5d and 18°C /30d samples from 3 experimental replicates. An asterisk represents significant difference compared to 28°C sample (P<0.05). **: P<0.01, ***: P<0.001. (PDF) [file pone.0160358.s003.pdf]

**A**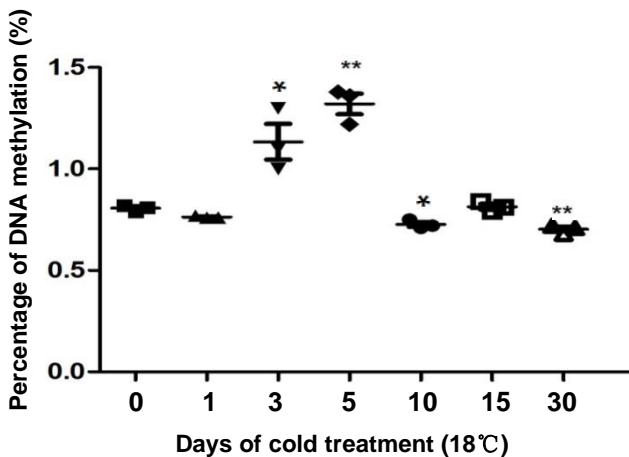**B**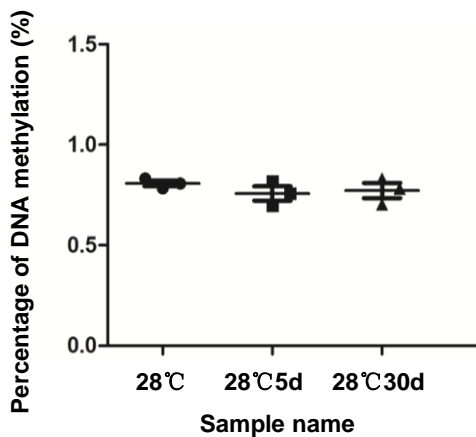

Supplement: S4 Fig — Genomic DNAs were isolated from cells cultured at 18°C (A) or 28°C (B) for indicated times. Quantitation assay of global genomic DNA methylation was performed with MethylFlash Methylated DNA 5-mC Quantification Kit. Percentage of DNA methylation was calculated according to the protocol. Error bars represent standard deviations (SD) (n = 3). An asterisk represents significant difference of signal compared to 28°C sample (P<0.05). **: P<0.01. ***: P<0.001. (PDF) [file pone.0160358.s004.pdf]

**A**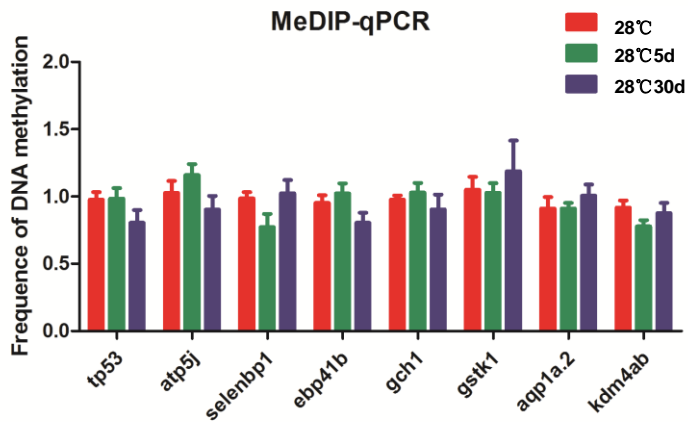**B**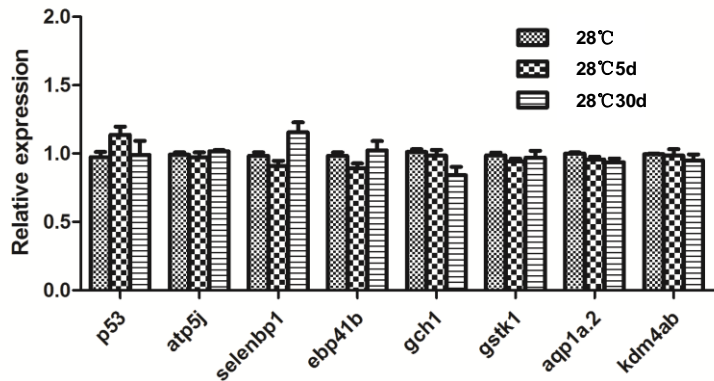

Supplement: S5 Fig — ZF4 cells were cultured at 28°C for 5 or 30 days, then MeDIP-qPCR (A) and RT-qPCR (B) were performed to detect DNA methylation levels in promoter regions and mRNA levels of indicated genes. Frequency of DNA methylation and relative expression were carried out by the comparative threshold cycle (CT) method. Statistical analysis was performed using GraphPad Prism 5 software. The student t test was used on measurements from 3 experimental replicates. Error bars represent standard deviations (SD) (n = 3). An asterisk represents significant difference compared to 28°C sample (P<0.05). **: P<0.01, ***: P<0.001. (PDF) [file pone.0160358.s005.pdf]
