# Supplementary material for: Variation of DNA Methylome of Zebrafish Cells under Cold Pressure
Source: PLoS One. 2016 Aug 5;11(8):e0160358. doi: 10.1371/journal.pone.0160358 (PMC4975392; doi:10.1371/journal.pone.0160358)
Supplement: S2 Fig — Genomic DNAs from different samples were bisulfite converted and amplified with specific primers for esrra (A) and cacng6a loci (B). Ten individual clones were sequenced for each sample. IGV images created from MeDIP-seq data for esrra locus are shown in Fig A. Black dots present methylated CpG and circles present un-methylated CpG. Percentage of relative methylated CpGs is shown in the figure. (PDF) [file pone.0160358.s002.pdf]

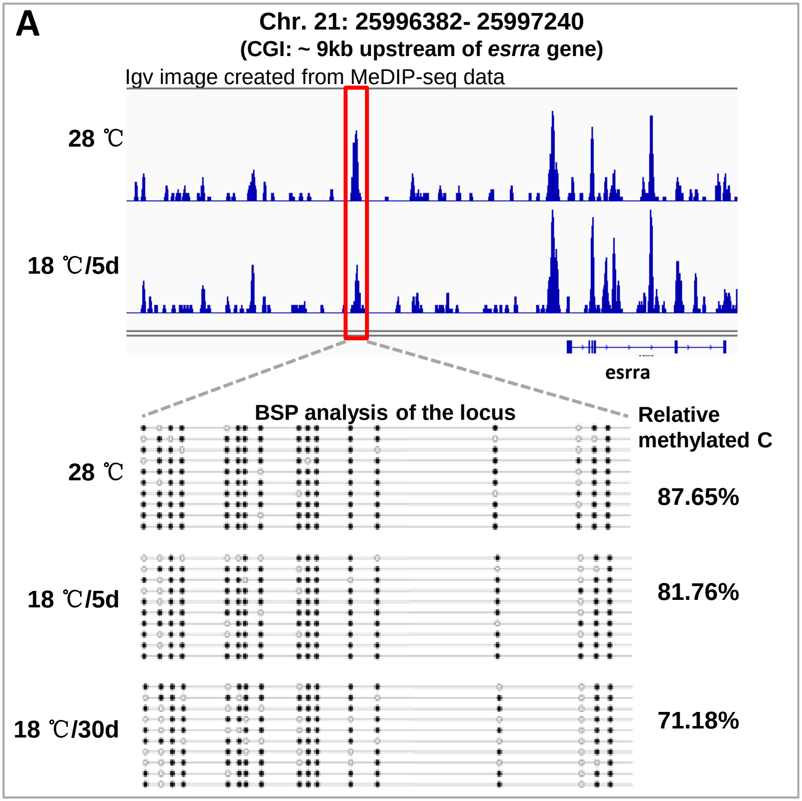
A
Chr. 21: 25996382- 25997240
(CGI: ~ 9kb upstream of esrra gene)
Igv image created from MeDIP-seq data
28 ℃
18 ℃/5d
esrra
BSP analysis of the locus
Relative methylated C
28 ℃
87.65%
18 ℃/5d
81.76%
18 ℃/30d
71.18%

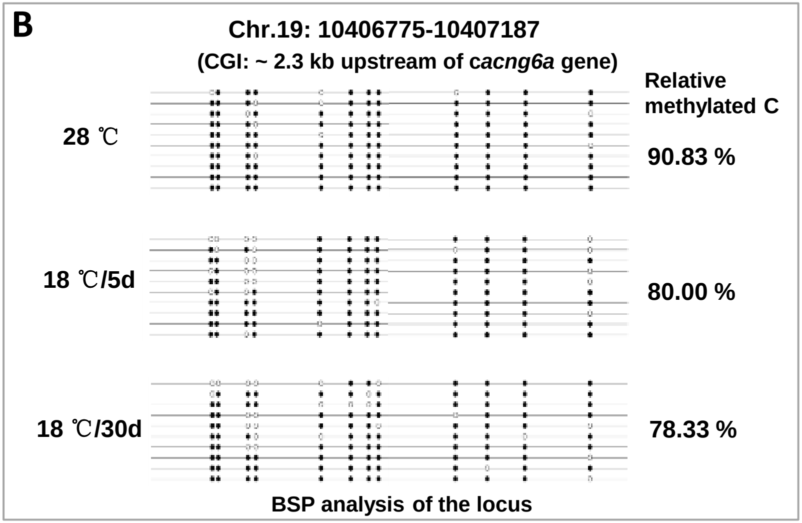
B
Chr.19: 10406775-10407187
(CGI: ~ 2.3 kb upstream of cacng6a gene)
Relative methylated C
28 ℃
90.83 %
18 ℃/5d
80.00 %
18 ℃/30d
78.33 %
BSP analysis of the locus
